# Selecting for Feed Efficient Cows Will Help to Reduce Methane Gas Emissions

**DOI:** 10.3389/fgene.2022.885932

**Published:** 2022-05-26

**Authors:** Coralia Ines Valentina Manzanilla-Pech, Rasmus Bak Stephansen, Gareth Frank Difford, Peter Løvendahl, Jan Lassen

**Affiliations:** ^1^ Center for Quantitative Genetics and Genomics, Aarhus University, Aarhus, Denmark; ^2^ Department of Animal and Aquacultural Sciences, Faculty of Biosciences, Norwegian University of Life Sciences, As, Norway; ^3^ Viking Genetics, Assentoft, Randers, Denmark

**Keywords:** feed efficiency, methane emission, residual methane, methane intensity, dairy cattle

## Abstract

In the last decade, several countries have included feed efficiency (as residual feed intake; RFI) in their breeding goal. Recent studies showed that RFI is favorably correlated with methane emissions. Thus, selecting for lower emitting animals indirectly through RFI could be a short-term strategy in order to achieve the intended reduction set by the EU Commission (-55% for 2030). The objectives were to 1) estimate genetic parameters for six methane traits, including genetic correlations between methane traits, production, and feed efficiency traits, 2) evaluate the expected correlated response of methane traits when selecting for feed efficiency with or without including methane, 3) quantify the impact of reducing methane emissions in dairy cattle using the Danish Holstein population as an example. A total of 26,664 CH_4_ breath records from 647 Danish Holstein cows measured over 7 years in a research farm were analyzed. Records on dry matter intake (DMI), body weight (BW), and energy corrected milk (ECM) were also available. Methane traits were methane concentration (MeC, ppm), methane production (MeP; g/d), methane yield (MeY; g CH_4_/kg DMI), methane intensity (MeI; g CH_4_/kg ECM), residual methane concentration (RMeC), residual methane production (RMeP, g/d), and two definitions of residual feed intake with or without including body weight change (RFI1, RFI2). The estimated heritability of MeC was 0.20 ± 0.05 and for MeP, it was 0.21 ± 0.05, whereas heritability estimates for MeY and MeI were 0.22 ± 0.05 and 0.18 ± 0.04, and for the RMeC and RMeP, they were 0.23 ± 0.06 and 0.16 ± 0.02, respectively. Genetic correlations between methane traits ranged from moderate to highly correlated (0.48 ± 0.16–0.98 ± 0.01). Genetic correlations between methane traits and feed efficiency were all positive, ranging from 0.05 ± 0.20 (MeI-RFI2) to 0.76 ± 0.09 (MeP-RFI2). Selection index calculations showed that selecting for feed efficiency has a positive impact on reducing methane emissions’ expected response, independently of the trait used (MeP, RMeP, or MeI). Nevertheless, adding a negative economic value for methane would accelerate the response and help to reach the reduction goal in fewer generations. Therefore, including methane in the breeding goal seems to be a faster way to achieve the desired methane emission reductions in dairy cattle.

## Introduction

Methane (CH_4_) contributes substantially to global warming, being the second most important greenhouse gas (GHG) after carbon dioxide (CO_2_; Knapp et al., 2014). Enteric fermentation by ruminants contributes to 44.3% of the global livestock emissions (FAO, IFAD, UNICEF, WFP, AND WHO, 2018). Methane is a by-product of the enteric (microbial) fermentation of carbohydrates in the reticulum-rumen of farm animals (Gray et al., 1951) and represents 8–9% of feed energy losses (Olijhoek et al., 2020). Furthermore, CH_4_ gas has been classified as a short-lived air pollutant (Tong et al., 2015), making its reduction a possible solution for global warming in the short term. Reducing enteric CH_4_ emissions in ruminants is imperative, given the commitment by the EU Commission (2021) to reduce GHG by 55% in 2030 and become neutral by 2050. Over the past decade, the scientific community has investigated different paths to reduce CH_4_ emissions, through different scientific disciplines, such as animal nutrition, physiology, management and genetics (de Haas et al., 2011; Waghorn and Hegarty, 2011; Alcock et al., 2015; Pickering et al., 2015). Some of the approaches include a) feed additives to reduce emissions (nutrition), b) identifying lower emitting animals at the same level of production (genetics), c) improving animal health, replacement of animals, and manure management, and d) reducing the consumption of animal products. Nevertheless, the advantage of genetics is that the reductions in CH_4_ are cumulative through generations and are permanent. Still, the combined sum of the strategies could help to reduce methane emissions faster and on time according to the EU regulations.

Enteric CH_4_ was an expensive and labor-intensive trait to collect with the traditional methods as respiration chambers; therefore, it was scarcely recorded until the appearance of new cheaper and easier methods (Garnsworthy et al., 2019). Given this lack of data, there is no common agreement on which traits should be included in the breeding goal trait in genetic/genomic selection (de Haas et al., 2017). The standard trait for methane emissions is methane production (MeP; g/d), as this is the trait measured by the most accepted method to measure CH_4_: respiration chambers. One of these new cheaper and easier methods, the “sniffers” (instrument measuring CH_4_ in breath by burps), is becoming more popular. These methods, though, measure CH_4_ as concentration (in ppm; MeC) not MeP. For this reason, some authors have used methane concentration as trait (López-Paredes et al., 2020; Sypniewski et al., 2021; Manzanilla-Pech et al., 2022) as it is a direct measurement from some recording instrumentation such as sniffers. However, this raw phenotype does not account for the size (weight), production or feed intake of the animal. Furthermore, animal nutritionist and farmers (Dijkstra et al., 2011; van Lingen et al., 2014) prefer definitions that express emission in terms of CH_4_ per kg of output, as CH_4_ intensity (MeI = g CH_4_/kg milk), or input like methane yield (MeY = g CH_4_/kg feed). However, selecting for a ratio trait present some statistical disadvantages like strong correlations with the denominator, in this case represented by economically important trait(s) already present in the breeding goal. Additionally it presents difficulty of interpretation of the selection response due to antagonism between the response in the numerator and the denominator (Berry and Crowley, 2013). For this reason, geneticists have suggested a residual trait (Hayes et al., 2016), adopting the concept of residual feed intake (RFI; Berry and Crowley, 2013)This approach creates a trait independent of production and weight, with the purpose of selecting for more feed efficient animals without affecting milk production. Several definitions of residual methane have been suggested, correcting or adjusting methane for production, feed, and weight (Manzanilla-Pech et al., 2016; Manzanilla-Pech et al., 2021; Richardson et al., 2021). Again, there is limited agreement on which residual trait should be used. Furthermore, this raw phenotype does not account for the size (weight), production, or feed intake of the animal. For this reason, it could be an option to use a residual methane trait (RMeC) that would help to account for milk production (energy corrected milk, ECM) and body weight (BW) of the animal in comparison to MeC that only provides a concentration. Another residual trait suggested could be RMeP that would help to reduce the induced covariance structure with MeP, ECM, and BW, given the use of these traits in the calculation of MeP (Madsen et al., 2010).

Given that feed costs represent a large proportion of the total costs of production and there has been a large interest in improving feed efficiency. In the last decade, several countries (i.e., Netherlands, Australia, United States, Denmark, United Kingdom) have included feed efficiency in their breeding goal through some index called saved feed or feed saved (Veerkamp et al., 2013; Pryce et al., 2015; Andersen et al., 2020; Holstein-USA, 2021; Li et al., 2021). Residual feed intake has been proposed as a proxy trait for feed efficiency in several species including cattle, pig, and poultry (Veerkamp et al., 2013; Sypniewski et al., 2021). Traditionally, feed efficiency can be defined as the difference between the actual and predicted intake. Moreover, feed efficiency is also related to energy balance (Pickering et al., 2015), defined as the difference between the energy a cow expends for milk production, maintenance, growth, and reproduction and the energy a cow gains from the intake of nutrients (Alcock et al., 2015). An unwanted effect of an increased negative energy balance is known to adversely affect fertility, so it remains important to adjust RFI for body weight changes to avoid this effect. Furthermore, methane emissions represent a gross energy loss from feed intake up to 12% (Gerber et al., 2013). Therefore, improving feed efficiency in cattle is also expected to help lower methane emissions (de Haas et al., 2017; Garnsworthy et al., 2019; Olijhoek et al., 2020). However, it has not been analyzed further what the impact of selecting for feed efficiency could have on lowering methane emissions. Therefore, it is required quantifying the possible reductions of methane as correlated response, when selecting for feed efficiency.

The objectives of this study were to 1) estimate genetic parameters for six CH_4_ traits (MeP, MeC, MeI, MeY, RMeP, RMeC), including genetic correlations between methane traits, production, and feed efficiency traits, 2) evaluate the expected correlated response of methane traits when selecting for feed efficiency with or without including methane, 3) quantify the economic impact of reducing methane emissions in Holstein dairy cattle in Denmark as an example.

## Materials and Methods

### Methane Data Collection and Editing

Measurements of CH_4_ and CO_2_ on 650 Holstein cows recorded between 2013 and 2020 at the Danish Cattle Research Center (DCRC, Tjele, Denmark) were available. Data have been partially described previously by Zetouni et al. (2018), Difford et al. (2020), and Manzanilla-Pech et al. (2020). Methane breath concentration (CH_4_ in parts per million, ppm, referred to as MeC) was measured by the non-dispersive infrared CH_4_ sensor (Guardian NG, Edinburgh Instruments Ltd., Livingston, United Kingdom), and in parallel, CO_2_ was measured using the same technique (Gascard, Edinburgh Instruments Ltd., Livingston, United Kingdom) installed in each of the three automatic milking stations (AMS). Equipment details, technical specification as sensor calibration and management of the raw data from thresholds for ambient background, and a cow head-lifting algorithm are reported in Difford et al. (2016). Cows were part of several nutritional experiments, and diets included primarily rolled barley, corn silage, grass clover silage, rapeseed meal, and soybean meal. The DCRC barn is a loose housing system with access to AMS (DeLaval International AB, Tumba, Sweden). Weekly records on ECM, BW, and DMI were available from 960 primiparous cows from the research farm (DCRC, Foulum, Denmark), most of them during the same period of time that methane concentration was measured, including cows with methane records and its contemporary relatives. Cows were fed with automated feeders (Insentec, RIC system, Marknesse, the Netherlands). Body weight was measured automatically at each milking and averaged per week (Li et al., 2017). The AMS was fitted with a weighing platform (Danvaegt, Hinnerup, Denmark) that recorded BW at each milking. ECM was calculated using the following formula (Sjaunja et al., 1991):

ECM (kg) = 0.25 Milk (kg) + 12.2 Fat content (kg) + 7.7 Protein content (kg). (1)

Weekly averages for CH_4_ and CO_2_ records were calculated to match the weekly records of ECM and BW available to calculate MeP. Data were filtered to only include the weekly averages, where a maximum of 3 days was allowed to be missing within a week, and individual cows required a minimum of three weekly measurements to be retained for further analysis. After editing, 26,664 weekly MeC records from 647 Danish Holstein cows and 19,123 MeP records from 575 from Holstein cows were analyzed. Natural logarithm (ln) transformation was applied, as MeC was not normally distributed, and this was multiplied by 100 to avoid problems with the scale of the other traits. Methane production was calculated as follows using the formula of (Madsen et al., 2010) based on heat producing units (HPU):

$${\text{CH}}_{4}\;\left(\text{L/d}\right)\;=\;\left({\text{CH}}_{4}\text{/}{\text{CO}}_{2}\right)\text{ x }180\text{ x }24\text{ x HPU,}$$ (2)

where

$$\text{HPU }=\;5\text{.}6{\text{ BW}}^{0.75}\;\left(\text{MBW}\right)\;+\text{ 22 ECM }+\;1{\text{.}6\text{ x }10-5\text{ × }\left(\text{number of days in pregnancy}\right)}^{3}.$$ (3)

Secondly, converting CH_4_ in L/d to g/d using the formula:

$$\text{MeP }={\text{CH}}_{4}\text{g/d }=\text{ Density ×}{\text{CH}}_{4}\left(\text{L/d}\right)\text{,}$$ (4)

where the density of CH_4_ at 20°C = 0.668 g/L.

Additionally, four methane traits that account for production level and weight were calculated: methane yield (MeY) was defined as MeP divided by DMI, and methane intensity (MeI) was calculated using MeP divided by ECM. Residual methane concentration (RMeC) was the residual of the partial regression of MeC on ECM and MBW, whereas RMeP was the residual of the partial regression of MeP on ECM and MBW along with fixed effects described in the model (5). Furthermore, residual feed intake 1 (RFI1) was the residual of the partial regression of DMI on MBW and ECM (according to the two-step RFI from Tempelman et al. (2015), along with fixed effects described in the model (5). An additional residual feed intake (RFI2) was based on the partial regression of DMI on MBW and ECM including body weight change (∆BW).

### Variance Component Estimation

For each trait (MeP, MeC, MeI, MeY, RMeP, RMeC), variance components were estimated using the AI-REML algorithm with the DMU software (Version 6, Release 5.4; (Madsen and Jensen, 2014)). Genetic and phenotypic correlations were estimated through pairwise bivariate analyses between the traits. A pedigree containing the identification of the cow, sire, and dam with 11,778 animals (3,024 sires and 7,754 cows) after pruning in the relationship matrix was used, with an average of 10 generations.

The model used to estimate the variance components was

$$y_{\mathrm{ijklmn}}\mathrm{=\mu +EY}S_{i}\mathrm{+ LAC}T_{\mathrm{WEEK}j}\mathrm{+ PA}R_{k}\left(\mathrm{ACC}\right)+a_{l}\mathrm{+ p}e_{m}+e_{\mathrm{ijklmn}},$$ [5]

where $y_{\mathrm{ijklmn}}\;$ is the phenotype for MeC, MeP, MeY and MeI; μ is the mean; *EYS* is the fixed effect i^th^ for experiment-year-season (115 classes); *LACT*_*WEEK*_ is the fixed effect j^th^ for a week of lactation (44 classes); *PAR* is the fixed effect for the k^th^ parity number (1, 2, 3+); and ACC is the age of cow at calving in months as covariate. Random effects are as follows: a is the additive genetic effect l^th^ distributed as N (0, A $\sigma_{a}^{2}$ ), in which A is the relationship matrix and $\sigma_{a}^{2}$ is the genetic variance, pe is the permanent environmental effect m^th^ (within and across parities) distributed as N (0, I $\sigma_{pe}^{2}$ ), where I is an identity matrix and $\sigma_{pe}^{2}$ is the permanent environmental variance and e is the residual effect of $y_{\mathrm{ijklmn}}$ . The model for the residual traits (RMeC, RMeP, RFI1, RFI2) included only the mean, additive genetic effect, permanent environmental effect, and residual effect, as the fixed effects have been accounted for in the calculation of the residual trait.

### Correlated Response of Selection in Past, Current, and Future Scenarios

Given that currently RFI is part of the saved feed index (Andersen et al., 2020) and it has been included in the net total merit (NTM) since 2020, an interesting question is to quantify the reduction on methane traits given its favorable correlation with RFI. The definition of saved feed in the Nordic countries was described by Sørensen et al. (2018) as: EBV (saved feed) = EBV (maintenance) + EBV(metabolic efficiency), where maintenance is MBW and metabolic efficiency is RFI. Therefore, we developed three selection indexes: a) MeP, b) RMeP, and c) MeI. Methane production was chosen, as it is the most commonly used trait in terms of methane emissions in dairy cattle. Residual methane was proposed in this study as candidate trait to be included in the breeding goal given its independency with production and weight. Methane intensity was included due to its popularity among some sectors and as exercise to quantify the possible CH_4_ reduction in terms of the unit of product (milk). Within each index, there were three possible scenarios reflecting past, current, and future situations. Given the lack of information on the correlation between functional traits included in the NTM (e.g., fertility and longevity) and methane production, this exercise only focused on ECM, BW and RFI. Furthermore, all scenarios are assuming zero correlations with health traits based on Zetouni et al. (Gerber et al., 2013) (udder health = 0.06), and low to zero correlations with conformation traits (stature = 0.01, body depth = -0.03 and chest width = −0.20). Last, as the genetic correlation between RFI1 and RFI2 was 0.86, for the correlated response, we used RFI1 given its practicality in the calculation. Scenario zero (SC0) represents the impact on methane with the past situation, when selection was only for milk production (ECM); scenario one (SC1) represents the current situation with RFI included through saved feed in the breeding goal for Nordic countries; scenario 2 (SC2) represents a future situation where methane is included in the breeding goal. The scenario zero (base scenario; SC0) only included an economic value for ECM (−0.6). Additionally, scenarios 1 and 2 (SC1 and SC2) included an economic value for RFI of -0.2 euro, according to the results of Stephansen et al. (2021). Furthermore, scenario (SC2) has been divided into A and B with different economic values, A) −0.005 and B) −0.017 being for MeP and RMeP, respectively. These values were based on the status report for 2021 for Denmark (Klimarådet, 2022), where the price suggested is 1,500 Danish kroner per ton of CO_2_e, and we took two possible scenarios (EPA Agency, 2022) A) to a 100-year global warming potential where 1 kg CH_4_ = 25 CO_2_e, and B) to a 20-year global warming potential period where 1 kg CH_4_ = 84 CO_2_e. For MeI, the economic values were calculated as the trait definition, g MeP/kg of ECM being equal to -0.0083 for scenario 2A (−0.005/0.6) and −0.0283 (−0.017/0.6) for scenario 2B. The economic values for MeP and RMeP are given in euro per g/d, whereas the economic values for MeI are given in euro per g MeP/kg ECM; and ECM and RFI are given in euro per kg/d. All scenarios set economic values for MBW to zero. Genetic variances, correlations and heritabilities obtained from this study were used in the calculation of the expected responses of ECM and RFI and the correlated responses for MBW and MeP or RMeP. Furthermore, reliabilities of the EBVs were assumed at 0.81 (average reliability for ECM).

## Results and Discussion

Descriptive statistics for all traits (MeP, MeC, MeI, MeY, RMeP, RMeC, RFI1, RFI2) are presented in Table 1. The average for MeP was 337.9 g/d, whereas for MeI, it is 9.2 g CH_4_/kg ECM and for MeY, it is 15.4 g CH_4_/kg DMI. These averages are similar to the values previously reported by Lassen and Løvendahl (2016), Breider et al. (2019), Richardson et al. (2021), and Sypniewski et al. (2021).

**TABLE 1:** Descriptive statistics for methane, and efficiency traits for Danish Holstein cows.

Trait	Number of cows	Number of records	Unit	Mean	SD	Minimum	Maximum
MeP	575	19,126	g/d	337.9	86.2	77.3	598.7
MeC	647	26,664	log (ppm)*100	573.8	51.5	414.0	731.5
MeI	573	19,018	g CH_4_/kg of ECM	9.2	2.3	1.9	29.5
MeY	572	18,845	g CH_4_/kg of DMI	15.4	3.4	3.7	35.8
RMeP	511	9,511	g/d	−0.1	62.7	−310.7	271.4
RMeC	517	11,285	g/d	−0.6	45.3	−169.1	168.9
RFI1	955	31,839	g/d	0.0	1.8	−16.9	11.1
RFI2	955	14,842	g/d	0.0	2.2	−15.9	12.1

MeP = methane production, MeC = methane concentration, MeI = methane intensity, MeY = methane yield, RMeP = residual methane production on ECM and MBW, RMeC = residual methane concentration on ECM and MBW, RFI2 = residual feed intake on ECM and MBW, and RFI2 = residual feed intake on ECM, MBW, and BW.

### Heritabilities and Genetic Correlations

Estimated genetic, permanent environmental, residual variances, heritabilities, and repeatabilities for all traits are presented in Table 2. Heritabilities were moderate ranging from 0.13 ± 0.02 (RFI1) and 0.23 ± 0.06 (RMeC). Estimated heritability for MeP was 0.21 ± 0.05, which is consistent to the previously reported estimates (Lassen and Løvendahl, 2016; Pszczola et al., 2017; Breider et al., 2019). Likewise, heritability for MeI was 0.18 ± 0.04, similar to the values reported previously (Lassen and Løvendahl, 2016; Kandel et al., 2017; van Engelen et al., 2018).

**TABLE 2:** Estimated genetic (σ^2^_a_), permanent environmental (σ^2^_pe_), residual (σ^2^_e_) variances, heritabilities (h^2^), permanent environmental ratios (pe^2^) and repeatabilities (rep) with (SE) for methane, production, maintenance, and efficiency traits.

Trait	σ^2^_a_	σ^2^_pe_	σ^2^_e_	h^2^	pe^2^	rep
MeP	1,160.6	1,456.8	2,923.9	0.21 (0.05)	0.26 (0.04)	0.47 (0.04)
MeC	474.8	956.9	942.9	0.20 (0.05)	0.40 (0.05)	0.60 (0.04)
MeI	0.7	1.2	2.3	0.18 (0.04)	0.29 (0.04)	0.47 (0.04)
MeY	2.2	2.3	5.7	0.22 (0.05)	0.23 (0.04)	0.45 (0.04)
RMeP	629.7	827.5	2,384.9	0.16 (0.04)	0.21 (0.04)	0.37 (0.04)
RMeC	479.5	716.2	881.3	0.23 (0.06)	0.34 (0.05)	0.57 (0.05)
RFI1	0.47	0.59	2.57	0.13 (0.02)	0.16 (0.02)	0.29 (0.02)
RFI2	0.98	1.06	3.25	0.18 (0.03)	0.20 (0.02)	0.38 (0.02)
ECM	26.9	9.1	21.1	0.47 (0.03)	0.16 (0.03)	0.63 (0.03)
MBW	34.7	26.1	11.7	0.48 (0.04)	0.36 (0.03)	0.84 (0.03)

MeP = methane production, MeC = methane concentration, MeI = methane intensity, MeY = methane yield, RMeP = residual methane production on ECM and MBW, RMeC = residual methane concentration on ECM and MBW, RFI1 = residual feed intake on ECM and MBW, RFI2 = residual feed intake on ECM, MBW, and BW.

Estimated genetic (r_g_) and phenotypic correlations (r_p_) between the six methane traits (MeP, MeC, MeI, MeY, RMeP, RMeC) are presented in Table 3. All methane traits were moderate to highly positive phenotypically correlated to each other, ranging from 0.48 ± 0.02 (MeI-MeC) to 0.98 ± 0.00 (RMeC-MeC). Genetic correlations of MeC (the phenotypic raw trait) with the other methane traits ranged from 0.48 ± 0.16 (MeI) to 0.71 ± 0.12 (MeP), whereas r_g_ between MeP (the reference trait) with the other methane traits ranged from 0.48 ± 0.16 (MeI) to 0.82 ± 0.07 (RMeP). Furthermore, RMeP was highly (above 0.8) genetic and phenotypically correlated with all the traits except for MeC; this could represent an advantage when this trait is added to the breeding goal given that it could represent all the other methane phenotypes. For example, as for farmers and nutritionists, MeI and MeY are important, and the correlations between RMeP and MeI and MeY are 0.85 and 0.88, respectively, and RMeP could be used as a proxy for them.

**TABLE 3:** Estimated genetic (below diagonal) and phenotypic correlations (above diagonal) with (SE) between methane traits.

Trait	MeP	MeC	MeI	MeY	RMeP	RMeC
MeP	—	0.63 (0.02)	0.51 (0.02)	0.84 (0.01)	0.81 (0.01)	0.52 (0.02)
MeC	0.71 (0.12)	—	0.48 (0.02)	0.59 (0.02)	0.63 (0.01)	0.98 (0.00)
MeI	0.48 (0.16)	0.48 (0.16)	—	0.59 (0.02)	0.85 (0.01)	0.56 (0.02)
MeY	0.77 (0.07)	0.58 (0.14)	0.58 (0.14)	—	0.84 (0.01)	0.55 (0.02)
RMeP	0.82 (0.07)	0.70 (0.11)	0.85 (0.07)	0.88 (0.05)	—	0.65 (0.01)
RMeC	0.77 (0.08)	0.69 (0.12)	0.84 (0.08)	0.95 (0.02)	0.98 (0.01)	—

MeP = methane production, MeC = methane concentration, MeI = methane intensity, MeY = methane yield, RMeP = residual methane production on ECM and MBW, RMeC = residual methane concentration on ECM and MBW, RMeP = residual methane production on ECM, MBW, and DMI..

Estimated genetic and phenotypic correlations between methane traits and efficiency traits are presented in Table 4. Genetic correlations between RFI1 and methane traits were low to moderate, positively correlated for all traits, ranging from 0.16 ± 0.19 (MeY) to 0.65 ± 0.13 (MeP), whereas r_g_ between RFI2 (including ∆BW) ranged from 0.05 ± 0.20 (MeI) to 0.76 ± 0.09 (MeP). These positive genetic correlations are favorable when selecting for RFI, given that the goal is to reduce RFI; this will also reduce methane emissions (in different magnitudes depending on the trait). However, for some of the traits (MeC, MeI, MeY) due to the large standard errors, these genetic correlations were not different from zero. The highest correlations with RFI (1 and 2) were found with MeP and RMeP. Nevertheless, for MeP, it would be difficult to increase milk and decrease methane at the same time given the high genetic correlation between MeP and ECM (0.79). Thus, RMeP could be suggested as a trait to include in the breeding goal given its low to zero correlation with ECM (0.12) and BW (0.06), meaning that this trait is almost independent of these traits and we could decrease methane without compromising milk production or the weight of cows. However, this suggestion is based on our results, including high and positive genetic correlations between RMeP, MeP, and RFI; further analyses with more data would be recommended to confirm these results.

**TABLE 4:** Estimated genetic (r_g_) and phenotypic (r_p_) correlations with (SE) between methane traits and production, maintenance, and efficiency traits.

	Trait	MeP	MeC	MeI	MeY	RMeP	RMeC
r_g_	RFI1	0.65 (0.13)	0.34 (0.18)^a^	0.28 (0.19)^a^	0.16 (0.19)^a^	0.48 (0.15)	0.33 (0.15)
	RFI2	0.76 (0.09)	0.32 (0.17)^a^	0.05 (0.20)^a^	0.35 (0.16)^a^	0.47 (0.15)	0.36 (0.17)
	ECM	0.79 (0.05)	0.35 (0.14)	−0.54 (0.11)	0.42 (0.12)	0.12 (0.16)^a^	−0.11 (0.16)^a^
	MBW	0.32 (0.14)	0.26 (0.17)^a^	0.37 (0.14)	−0.11 (0.15)^a^	0.06 (0.16)^a^	0.20 (0.17)^a^
r_p_	RFI1	0.17 (0.02)	0.12 (0.02)	0.19 (0.02)	−0.23 (0.02)	0.22 (0.02)	0.13 (0.02)
	RFI2	0.45 (0.02)	0.23 (0.02)	−0.09 (0.03)	0.01 (0.03)^a^	0.16 (0.02)	0.09 (0.03)
	ECM	0.58 (0.02)	0.22 (0.03)	−0.41 (0.02)	0.30 (0.03)	−0.01 (0.03)	−0.02 (0.03)^a^
	MBW	0.19 (0.03)	−0.01 (0.03)^a^	0.01 (0.03)^a^	0.04 (0.03)^a^	0.20 (0.03)	0.00 (0.03)^a^

MeP = methane production, MeC = methane concentration, MeI = methane intensity, MeY = methane yield, RMeP = residual methane production on ECM and MBW, RMeC = residual methane concentration on ECM and MBW, RFI1 = residual feed intake on ECM and MBW, RFI2 = residual feed intake on ECM, MBW, and BW.

a Correlations not different from zero based on two SE.

### Best Candidate Trait to be Included in the breeding Goal

As stated before, many countries have opted to use sniffers to record methane emissions, given the practical ease of installation in automated milking stations, bringing an additional phenotype called CH_4_ breath concentration (MeC) (Madsen et al., 2010; Calderón-Chagoya et al., 2019; Sypniewski et al., 2019; Difford et al., 2020). However, to calculate MeP from CH_4_ (and CO_2_), ECM and BW are needed (Madsen et al., 2010), leading to an artificially induced covariance structure between traits resulting in high correlations between MeP and ECM and BW (Manzanilla-Pech et al., 2020; Manzanilla-Pech et al., 2022). This induced covariance could be removed by the calculation of adjusted traits as residual traits. Residual methane concentration calculation had the purpose of having a trait that does not involve MeP (but MeC) accounting for the weight and production of the animal. However, RMeC had lower genetic correlations with MeP and RFI in comparison with RMeP. Therefore, the use of RMeP seems to be the most plausible for including in a selection index, especially since its genetic correlations with the other methane traits are high and positive, ranging from 0.70 (MeC) to 0.98 (RMeC), and the genetic correlation with MeP is 0.82 and RFI is 0.48. Similarly, Richardson et al. (Richardson et al., 2021) proposed a residual methane trait adjusted only by ECM, as candidate trait for the inclusion in routine genetic evaluation, based on its practicality of recording ECM in a pasture-based system. Breath concentration traits (MeC and RMeC) could have some benefits being the raw unadulterated trait (MeC) and not being “modified” using traits as ECM and MBW. However, given that the unit of MeC and RMeC is log(ppm), the interpretation of the addition to the breeding goal of any of these traits will not be straightforward as other traits are in kg/d. Additionally, farms and AMS designs have a great impact on the ppm values, which makes a fair comparison of phenotype difficult and reduce its suitability to be elected as a candidate methane trait. Furthermore, MeI and MeY are popular among some sectors (nutritionists, farmers, and livestock farming systems) as they are presented in terms of the unit of milk produced or the unit of feed consumed. Despite that ratio traits (MeI and MeY) are not the preferred candidate trait to be included in the breeding goal given the problems previously stated, this does not mean that MeI or MeY cannot be improved. By selecting on other methane traits highly correlated with MeI or MeY, it is possible to improve these traits. Gerber et al. (2011) acknowledged MeI as a proxy for system productivity and concluded that even if MeP increases with higher yields, MeI declines as animal productivity increases. This could dilute the effect of the emissions in one cow over a larger volume of milk produced, in comparison to requiring the number of lower milk producing cows needed to produce the same amount of milk under a low or fixed level production system, which would inevitably produce more CH_4_ than a single cow. In conclusion, RMeP is a suitable candidate trait to be included in the breeding goal, given its high correlations with other methane traits and that it has the same advantages as MeP without compromising the genetic correlations with production and maintenance traits. The residual methane production allows for disentanglement of the differences between cows similar in production and weight with respect to their methane production. Still, some traits as MeI would be useful when explaining or comparing the amount of methane produce respecting the amount of milk produced.

### Correlated Response of Methane When Selecting for Feed Efficient Cows

Correlated response to selection for MeP, RMeP, and MeI using ECM, MBW, and RFI is presented in Table 5. Most scenarios for MeP and RMeP presented a positive response to selection, which can be translated as an increase on methane emissions. The exception was scenario SC2B for RMeP where the response was negative (−0.88), meaning an actual reduction on the methane emissions. Unlike, MeI (g CH_4_/kg ECM) decreased in all scenarios. The scenario zero (SC0) showed a scenario where the main trait to select was ECM. In SC0, the correlated response of MeP was an increase of 24.22 g/d, whereas for RMeP, it was 2.71 g/d; this would represent an increase of 7% with respect to the current average (337.9 g/d) for MeP. For MeI (in SC0), the correlated response was -0.41 g of CH_4_/kg of ECM. Scenario 1 (SC1), where RFI is included, observed a reduction in MeP of 0.5 g/d and for RMeP of 0.4 g/d. For MeI (SC1), the correlated response was -0.42 g of CH_4_/kg of ECM, which represents 1 g less per kilogram of milk in comparison to SC0. These reductions are per animal per generation and they are cumulative. Scenario 2, where we included an economic value for methane emissions at two different levels, reduced MeP by 1.43 g/d (SC2A) up to 4.16 g/d (SC2B) compared to SC0, whereas RMeP reduced by 1.37 g/d (SC2A) up to 3.59 g/d (SC2B). These reductions in SC2 represent CH_4_ reductions of 6–17% compared to SC0 independently of the trait. For MeI (SC2), the correlated response was −0.43 g of CH_4_/kg of ECM, which represent 2 g less per kilogram of milk in comparison to SC0. Translating these values to kg per cow per lactation, for SC2A these reductions are between 0.44 (MeP) and 0.42 kg/cow/year (RMeP), whereas for SC2B, these reductions are between 1.3 (MeP) and 1.1 kg/cow/year (RMeP). Similarly, Gonzalez-Recio et al. (2020) reported a decrease of 0.39 kg/cow per year when including methane in the breeding goal, resulting in a 20% reduction in 10 years. Additionally, there is an (negative) effect on the genetic gain of ECM when including RFI and greater when including methane. However, this possible reduction (deceleration in the increase) on the genetic gain of ECM is lower when including RMeP (2%) instead of MeP (5%) in SC2B. González-Recio et al. (2020) have also reported a possible reduction of 10–18% in the genetic gain for production traits as a collateral effect of including methane in the breeding goal.

**TABLE 5:** Correlated response to selection for MeP, RMeP, and MeI using ECM, MBW, and RFI.

Trait	Scenarios	Input economic values (€)				Output response			
		ECM	RFI	MBW	MeP	ECM	RFI	MBW	MeP
MeP	SC0	0.6	0	0	0	4.67	0.30	0.05	24.22
	SC1	0.6	−0.2	0	0	4.65	0.28	0.01	23.73
	SC2A	0.6	−0.2	0	−0.005	4.60	0.26	−0.09	22.79
	SC2B	0.6	−0.2	0	−0.017	4.42	0.22	−0.35	20.06
RMeP	SC0	0.6	0	0	0	4.67	0.30	0.05	2.71
	SC1	0.6	−0.2	0	0	4.65	0.28	0.01	2.27
	SC2A	0.6	−0.2	0	−0.005	4.64	0.26	0.02	1.34
	SC2B	0.6	−0.2	0	−0.017	4.58	0.23	0.05	−0.88
MeI	SC0	0.6	0	0	0	4.67	0.30	0.05	−0.41
	SC1	0.6	−0.2	0	0	4.65	0.28	0.01	−0.42
	SC2A	0.6	−0.2	0	−0.0083	4.65	0.27	0.00	−0.43
	SC2B	0.6	−0.2	0	−0.0283	4.65	0.27	0.00	−0.43

MeP = methane production, RMeP = residual methane production on ECM and MBW, MeI = methane intensity, RFI = residual feed intake on ECM and MBW, ECM = energy corrected milk, MBW = metabolic body weight (BW^0.75^), SC0 = scenario zero, only selecting for ECM, SC1 = scenario one, including and RFI and ECM, SC2n = scenario two, including ECM, RFI and methane, either MeP, RMeP with different economic values (A = -0.005, B = -0.017).

There are several interesting points in this analysis; first, that there is a reduction of methane emissions in SC1, meaning that in the current situation even if we do not add methane into the breeding goal, there is a reduction of methane emissions by including RFI in the breeding goal; given that methane is correlated with RFI. Several authors have pointed out previously that by selecting for RFI, we could reduce methane emissions (Hegarty et al., 2007; de Haas et al., 2011; Manzanilla-Pech et al., 2021). However, the physiological mechanism behind this reduction of methane by selecting for feed efficient animals remains unknown. Basarab et al. (2013) have previously summarized three hypotheses as to why lower RFIs have lower MeP or MeY; the first one states that efficient animals with lower feed energy intake at equal levels of production and BW have lower MeP. Second, one implicates that shorter retention time of digestion in the rumen is related with lower MeP, and a third one involves ruminal retention in combination with host response in microbial communities favoring digestibility. On the other hand, some nutritionist experiments have unsuccessfully attempted to prove a link between low RFI and low CH_4_ (Hegarty et al., 2007; Fitzsimons et al., 2013; McDonnell et al., 2016). As nutritional manipulations sometimes affect RFI and give results in directions opposite to genetic differences, likely as effects on the microbiome. However, the effects of additives on the microbiome are indeed interesting and they could be useful in combined strategies (genetics plus feed additives). Additionally, a number of animals limit most of the nutritional experiments and no genetic component is included, but several diets are, which could explain the results. These results (Hegarty et al., 2007; Fitzsimons et al., 2013; McDonnell et al., 2016) showed little evidence of a direct effect of RFI on ruminal CH_4_ emissions (g/day) and that differences observed are a reflection of the variance in DMI between animals (Kenny et al., 2018). Therefore, this topic needs to be further investigated to elucidate the physiological mechanism behind the links between methane reduction and feed efficiency.

Despite the reduction on the expected response (deceleration) of methane emissions by including RFI in the breeding goal, it seems that this reduction will not be enough to reach the intended global reductions of GHG at country and at the EU level (EU Commission, 2021). Therefore, additional (negative) weights should be included for methane traits to accomplish a desired reduction in the GHG emission from dairy cattle in the next 10 and 30 years. For this reason, including methane through one of its traits is a potential way to accelerate this reduction on methane emissions. The outcome of this analysis shows that including either MeP or RMeP in the breeding goal would be translated as a deceleration of the increase of methane emissions compared to the actual scenario, where no methane trait is included. However, the advantage of RMeP would be that this trait is independent of the production and body weight of the animal. For this reason, the effect of including an economic value for methane has smaller effect on the genetic gain of ECM, in comparison to that of MeP. This is the primary advantage of residual traits; in this case, RMeP has been corrected by ECM and MBW, thus having low or close to zero correlations with the production traits.

Furthermore, there has been an interest to express the reduction in MeI; in other words, in terms of the unit of CH_4_ per kg of ECM produced. In this study, we also included MeI as a candidate trait to visualize the correlated response of MeI when including RFI in the breeding goal. The response was favorable (negative), meaning that selecting for efficient cows will also have a positive impact by reducing methane (−0.41 to −0.43 g) for methane emitted by the unit of production (kg of ECM). As mentioned before, given the increase in milk production per cow, fewer cows are needed to produce the same amount of milk. So, independently of the amount of CH_4_ produced per cow, the MeI would be diluted by the higher yield, meaning that a high producing cow will have lower MeI than a low producing cow. In the last decade, as a result of selection on highly milk producing cows, MeI (g CH4/kg ECM) has shown a constant decrease (Moate et al., 2014; González-Recio et al., 2020). Therefore, the breeding objective could help to improve MeI but by including MeP (or RMeP) and ECM in the selection index and not MeI directly. Moreover, it is arbitrary how to include a ratio trait into the breeding goal, as it is unclear whether the response is established through changing the numerator, the denominator, or both.

### Sensitivity Analysis: Study Case Denmark

Based on the results of the selection index analysis, we quantified the economic impact of the reduction of methane possible when including either MeP or RMeP in the breeding goal. This exercise was done for Denmark, based on the Danish statistics publicly available (Klimarådet, 2022; Statbank Statistics Denmark, 2022). The volume of methane reduction was calculated as the difference between the two most extreme scenarios SC2B vs. SC0. For MeP, it was 4.16 g/d and for RMeP, it was 3.65 g/d; if we multiply this for 305 days per lactation, it gives us a reduction of 1.27 and 1.11 kg methane per year per cow, respectively. Multiplying these numbers for the total number of dairy cows in Denmark (Statbank Statistics Denmark, 2022) (550K) results in 612 and 698 ton CH_4_. Taking into account that the total amount of CH_4_ produced by dairy cattle in a year in Denmark was 86.59 kt (Albrektsen et al., 2021), this reduction barely represents 1%. However, if we translate this reduction of CH_4_ to cost, the importance of this exercise becomes clearer. As CH_4_ is usually expressed in a ton of CO_2_ equivalents (CO_2_e), to convert ton CH_4_ to ton CO_2e_ requires a multiplication of 25 (for a 100-year global warming potential where 1 kg CH_4_ = 25 CO_2_e) or for 84 (for a 20-year global warming potential period where 1 kg CH_4_ = 84 CO_2_e). For MeP, this would represent 17.4 K ton CO_2_e or 58.6 K ton CO_2_e depending on the period of global warming potential used, whereas for RMeP, this would represent 15.3 K ton CO_2_e or 51.4 K ton CO_2_e. Based on the previous calculation of 1,500 DKK (or €200) per ton CO_2_e according to Klimarådet (2022), this apparently small reduction at the cow level on methane emissions could be translated to a saved cost of 3.5–11.7 million € when using MeP and from 3.1 to 10.4 million € when using RMeP per year for the whole Danish dairy sector (1 € = 7.45 kroner). Our results are in agreement with the 25-year simulation in the Australian beef industry assuming an annual rate response of −0.08 kg DM/d, a cumulative CH_4_ reduction of 568,100 ton (22.7 K ton/year) was achieved, which represented €8.5 million/year in carbon credits (Alford et al., 2006).

In conclusion, residual methane production has shown potential benefits as candidate trait when selecting for reduced methane emissions, like being highly correlated with efficiency traits and weakly correlated with production. Additionally, residual methane production is highly correlated with the other methane traits, which is favorable when there is interest in more than one trait. Furthermore, selection index calculations showed that selecting for feed efficiency (RFI) has a positive impact on reducing methane emissions independently of the trait used; nevertheless, adding a negative economic value for methane would accelerate the response and help to reach the reduction goal in fewer generations. Therefore, including methane traits in the breeding goal shall be the preferable way to achieve the desired methane reductions in dairy cattle. Consequently, including methane in the breeding goal seems to be imperative in order to obtain the intended reduction of methane emissions in the coming years, despite the possible deceleration in the genetic gain of milk production. Additionally, if we translate the possible reductions of methane achieved by including methane in the breeding goal, this could lead to savings up to 11 million of Euros in 1 year to the dairy industry, just for Denmark.

## Data Availability

The datasets presented in this article were used under agreement with the Department of Animal Science from Aarhus University, https://anis.au.dk/en/ and hence, are not publicly available. Data are however available upon required agreement with all the partners involved and should be directed to the corresponding author coralia.manzanilla@qgg.au.dk.
